# Multimorbidity and health-related quality of life after tuberculosis treatment in Zimbabwe: A cross-sectional study with household comparators

**DOI:** 10.1371/journal.pgph.0007119

**Published:** 2026-09-01

**Authors:** Claire Jacqueline Calderwood, Edson T. Marambire, Trevor Musunzuru, Karlos Madziva, Fungai Kavenga, Eveline Muringi, Justin Dixon, Junior Mutsvangwa, Celia L. Gregson, Rashida Abbas Ferrand, Katherine Fielding, Katharina Kranzer

**Affiliations:** 1 National Heart and Lung Institute, Imperial College London, London, United Kingdom; 2 Clinical Research Department, London School of Hygiene & Tropical Medicine, London, United Kingdom; 3 The Health Research Unit Zimbabwe, Biomedical Research and Training Institute, Harare, Zimbabwe; 4 National Tuberculosis & AIDS Programme, Ministry of Health and Child Care, Harare, Zimbabwe; 5 Global Health and Ageing Research Unit, University of Bristol, Bristol, United Kingdom; 6 Department of Global Health and Development, London School of Hygiene & Tropical Medicine, London, United Kingdom; 7 Biomedical Research and Training Institute, Harare, Zimbabwe; 8 TB Centre, London School of Hygiene & Tropical Medicine, London, United Kingdom; 9 Division of Infectious Diseases and Tropical Medicine, University Hospital, LMU Munich, Munich, Germany; 10 German Centre for Infection Research (DZIF), Partner Site Munich, Munich, Germany; Federal University of Rio de Janeiro, BRAZIL

## Abstract

Chronic conditions both drive development of and are consequences of tuberculosis (TB). While there is growing awareness of chronic lung disease, data on other health conditions post-TB are limited. In this cross-sectional study in Zimbabwe, we offered adults with pulmonary TB an integrated health check at or after treatment completion, assessing uptake and yield. Members of the same households were also offered the intervention. Selecting household members without prior or current TB as a comparator, we calculated odds ratios (OR) for HIV, diabetes, hypertension, underweight, anaemia, symptoms of mental health disorders, self-reported memory difficulty, vision impairment, chronic lung disease and multimorbidity (two or more of the above); and compared health-related quality of life (HRQoL; EQ-5D value). Of 229 people with TB invited, 96 (42%) participated (median age 38 years, 54% men, median 356 days from diagnosis). Common reasons for non-participation were migration and work. Among people with TB, chronic conditions were common and, other than HIV, mostly undiagnosed. Ninety-one percent of people with recent TB had at least one chronic condition; almost two-thirds had two or more. Compared to 285 household comparators (median age 34 years, 71% female) and after adjustment for age and sex, people with recent TB were more likely to have at least one chronic condition (OR 2.30 [95% confidence interval 1.03–5.11]) but not multimorbidity (1.55 [0.86–2.78]). People with recent TB had greater odds of HIV (1.77 [1.03–3.04]), symptoms of mental health disorders (1.57 [0.96–2.56]), self-reported memory difficulty (2.13 [1.19–3.80]), impaired lung function (3.24 [1.46–7.20] among 39/92 people with TB and 155/285 household comparators with data available), and underweight (5.15 [2.28–11.61]) compared to household comparators without prior TB; hypertension was less common (0.40 [0.20–0.79]) and HRQoL was worse (p = 0.005). Uptake of chronic disease screening among people with recent TB was low, but prevalence of chronic conditions was high. Holistic approaches to treatment which identify and address these conditions are needed. The contribution of post-TB multimorbidity to morbidity and mortality among TB survivors should be included in global disease estimates. Whilst comparison to household controls aimed to reduce confounding by socio-economic status, a limitation of this approach is externality, whereby the health status of the person with TB influenced that of their household members.

## Introduction

Tuberculosis (TB) occurs as part of a multimorbidity complex with peak incidence among young adults [1]. Pre-existing health conditions including HIV, diabetes, undernutrition and chronic lung disease, and associated risk factor clusters (including alcohol, tobacco and other substance dependence), drive development of TB. TB, in turn, serves to entrench these conditions and their drivers – accelerating them and propelling development of new morbidities [2]. The long-lasting impacts of TB are increasingly recognised. Post-TB lung disease and other conditions reduce health-related quality of life (HRQoL) and increase hospitalisation and mortality years after TB treatment completion [3–5]. In 2020, approximately 155 million people alive globally were TB survivors; many now live with long term consequences of TB and, hence, half of the disability-adjusted life years attributable to TB occur after treatment completion [6–8].

The high prevalence of comorbidities among adults with TB at diagnosis has been described [9]; however, data on the post-TB period have generally focused on lung disease [10] with other chronic conditions and HRQoL outcomes infrequently considered. Specifically, whilst HRQoL appears to improve during the period of TB treatment, no studies in adults have compared HRQoL of TB survivors to community control groups [5,11]. Whilst in some cases, prevalent conditions during the post-TB period are likely to be similar to those at diagnosis (e.g., HIV), for others the acute biological and psychosocial effects of TB may exacerbate (e.g., diabetes, mental health, undernutrition) or mask (e.g., hypertension) chronic conditions. Further, other diseases may develop as late complications of TB or its treatment (e.g., vision impairment). A recent study in Kenya, Uganda, Zambia and Zimbabwe found high prevalence of HIV, undernutrition and hypertension (44%, 14% and 16%) among people assessed at the time of TB treatment completion, with 4% screening positive for mental health disorder and 3% for diabetes [12]. However, the lack of a comparator group in this study prevents the effects of TB being differentiated from pre-existing comorbidities and from the effects of the multitude of other adverse social, structural and environmental health determinants to which people with TB are exposed [13]. Comparison of non-respiratory comorbidities among people with prior TB to community controls was made in one study from Peru, with 5% prevalence of hypertension (vs 15% of community controls) and 11% prevalence of glycosuria (vs 5% of controls) [14]. Whilst communities often have similar socio-economic status, TB-affected households are often poorer than the community average and use of community controls may not fully account for differences in socio-economic determinants of health between TB-affected and other households.

As part of a study developing an integrated health check for members of TB-affected households, we offered a health check to people with TB, at or after TB treatment completion. In this paper, we sought to understand the uptake and yield of point-of-care multimorbidity screening for people with TB at or after treatment completion, and to compare the prevalence of chronic conditions, multimorbidity, and differences in HRQoL, between people with recent TB and without TB.

## Methods

### Ethical approvals and reporting guidance

Informed written consent was obtained from all participants. Ethical approval was granted by the Medical Research Council Zimbabwe (MRCZ/A/2618), and the ethical committee of London School of Hygiene & Tropical Medicine, United Kingdom (26625; parent ERASE-TB study, 22522). The study is reported in accordance with the Strengthening the Reporting of Observational Studies statement (Table A in S1 Appendix).

### Study design

This was a cross-sectional screening study among people with TB and their household members in Harare, Zimbabwe (Integrated multi-component health checks for TB-affected households; IMBA Hutano); and based within the wider Early risk assessment in paediatric and adult household contacts of confirmed tuberculosis cases by novel diagnostic tests (ERASE-TB) multi-country cohort study [15–17]. The primary aim of IMBA Hutano was to develop an integrated health check for TB household contacts; the results on uptake and yield of screening among household contacts are reported separately [15]. The focus of this paper is people with TB in the study households who were offered the same services at or after completion of their TB treatment (26 April 2022 to 11 March 2024). All were aged at least 18 years and had pulmonary TB (Xpert MTB/Rif Ultra medium-high positive at diagnosis) [16]. They were informed about the study at the time of their TB diagnosis and telephoned six months later. With consent, we asked family members participating in the parent study for updated contact details or to ask their relative to contact the study team in the event of being unable to contact individuals. People with recent TB were telephoned up to three times to invite them to participate. Screening included point-of-care HIV (for participants who did not already know their status) and HbA1c testing (A1c Care, SD Biosensor); and measurement of height, weight, blood pressure (digital sphygmomanometer with two or three measurements, five minutes apart) and haemoglobin (HemoCue); vision assessment (Peek Vision tests); spirometry (EasyOne, ndd Medical Technologies); and a locally-developed audio computer-assisted self-interviewing (ACASI) questionnaire on mental health (Shona Symptom Questionnaire [18]), use of alcohol (the alcohol use disorders identification test [AUDIT]), and other substances (the alcohol, smoking and substance involvement screening test [ASSIST]; details in Table B in S1 Appendix). We assessed potential self-reported memory difficulty using the question ‘In the past 12 months, have you had difficulty remembering things or following a story or conversation?’. This approach has not been validated locally; in fact no validated tools for memory impairment exist in our setting. We assessed HRQoL using a validated, interviewer-administered Shona translation of the 5-level EQ-5D version (EQ-5D-5L; EuroQOL) and physical activity using the International Physical Activity Questionnaire-Short Form (IPAQ-SF) [19,20].

### Statistical analysis

Data were analysed in R (version 4.3.1) and Stata (version 18).

We defined uptake of the health check among people with TB as the proportion of invited people who took up the intervention and the number of people completing each screening component, illustrated using a flow diagram. Yield was calculated as the proportion of people with a positive screening result, out of all those who participated in screening.

To describe the association between TB and chronic conditions or HRQoL, comparators were selected from the household contacts who participated in the same health check (20 April 2022 to 27 March 2023). People younger than 18 years, those with previous TB or co-prevalent or incident TB during the study, or those with missing data for screening tests were excluded.

We calculated the prevalence of each condition, having at least one condition (other than TB itself) and multimorbidity using a complete cases approach (i.e., excluding people who did not participate in screening). Chronic disease definitions followed international standards and established definitions (Table B in S1 Appendix). HIV was based on report of known HIV-positive status, being on anti-retroviral therapy (ART) or a positive HIV test. Underweight was defined as a body mass index (BMI) below 18.5 kg/m^2^, diabetes as HbA1c ≥ 6.5% or self-reported treated diabetes, anaemia as a haemoglobin concentration below the World Health Organization sex-specific threshold and hypertension as blood pressure ≥140/90 mmHg or self-reported treated hypertension. Quality-assured post-bronchodilator spirometry was used for analysis, with individuals with ‘good quality’ (A-C grade) spirometry included. Impaired lung function was defined as an obstructive, restrictive or mixed pattern, defined according to European Respiratory Society/American Thoracic Society technical standards and Global Lung Function Initiative (GLI) African American equations, previously shown to ‘best fit’ TB household contacts in our population; calculations based on GLI Global equations were used as a sensitivity analysis [21–23]. Multimorbidity was defined as the presence of two or more of HIV, diabetes, hypertension, symptoms of mental health disorder, self-reported memory difficulty, vision impairment, chronic lung disease, underweight or anaemia. HRQoL was described using crosswalk from EQ-5D-5L to the Zimbabwe EQ-5D-3L value set [24,25]. Analyses were *a priori* adjusted for age (four-level categorical variable: 18–29, 30–39, 40–49 and 50 years and older, unless otherwise stated) and sex; all models used robust standard errors to account for household-level clustering.

We first explored the differences in demographic characteristics and health behaviours between people with recent TB and household comparators, presented as proportions or medians and interquartile ranges (IQRs). To compare across groups, Wald p-values from logistic regression for the outcome of recent TB status by demographic and health behaviours as exposures were calculated. We then calculated odds ratios (ORs) with corresponding 95% confidence intervals (95% CI) for the effect of TB exposure status on the presence of each chronic disease, at least one condition, and multimorbidity, using logistic regression; primary analyses present models adjusted for age- and sex- (aOR, adjusted odds ratio), as a priori confounders of the association between TB and chronic conditions. From these models, we calculated age and sex-adjusted predicted probabilities of each outcome for people with and without TB, at the mean age category of people with TB in the study (30–39 years) and with balanced sex groups. As explorative analyses, we additionally adjusted for (a) HIV status and (b) BMI, as possible confounders of the relationship between TB and chronic conditions; and stratified by sex. We visually represented the overlap between chronic conditions using Euler plots, including the overlap with household poverty (i.e., income  < 2.15 United States Dollars [USD] per person per day [26]). No adjustment for multiple comparisons was made; estimates are interpreted on effect size and precision rather than thresholds; and stratified analyses are exploratory.

We compared HRQoL (EQ-5D) utility index and physical activity (metabolic equivalent of task [METS]) outcomes across TB exposure status using linear regression. The minimum clinically important difference (MCID) in HRQoL was defined as half the standard deviation (SD) of EQ-5D value in the overall study population [27]. As sensitivity analyses, we further considered HRQoL and physical activity as ordered categorical variables (HRQoL: no difficulty in any domain, at most slight difficulty in at least one domain, or moderate or higher difficulty in one or more domains; physical activity: low, moderate or high as per IPAQ-SF scoring protocol), modelling the relationship with ordinal logistic regression.

### Sample size

The study size was determined by the number of people with TB enrolled into the primary study. We aimed to offer health checks to all people with TB, at or after their TB treatment completion, assessing feasibility of doing so, and thus an *a priori* power calculation of the final study population was not performed. Table C in S1 Appendix details the power of the study to detect a range of effect sizes, with the current sample size and observed disease prevalence.

### Patient and public involvement

This study was developed, implemented and analysed with involvement of local researchers, policymakers and community, ensuring contextual relevance. This included presentations to our community advisory board and members of the National Tuberculosis/AIDS Programme at the protocol development stage, with feedback used to inform the study design. Results have been presented to the community advisory board and local and national stakeholders through national conferences and dedicated meetings.

## Results

### Study population

#### Adults previously diagnosed with TB.

**Fig 1:**
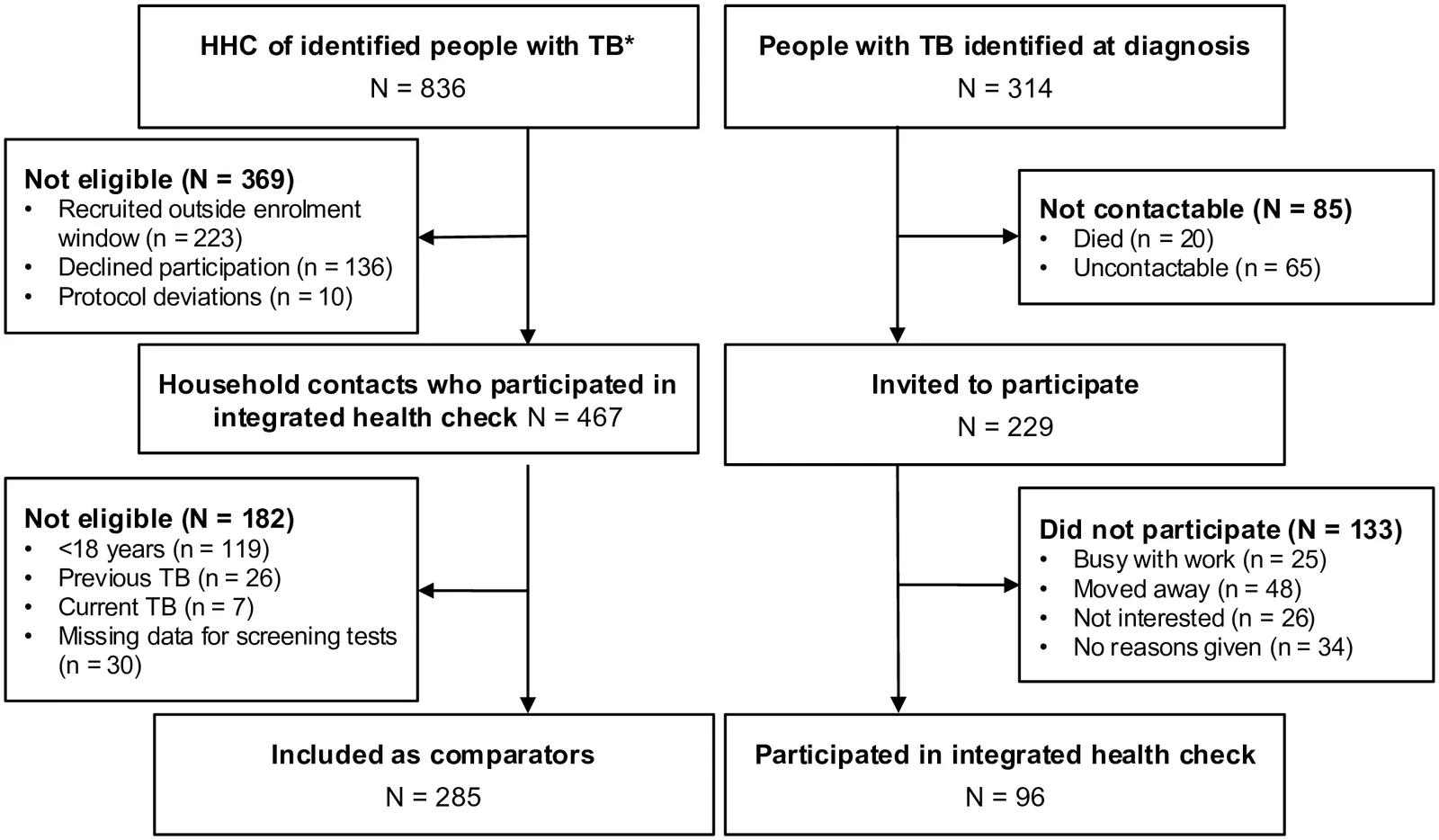
Flow chart illustrating study participation. * As per criteria for the ERASE-TB multi-country cohort study, household contacts (HHC) of adults (age ≥ 18 years) with microbiologically-confirmed, highly-infectious (Xpert MTB/Rif Ultra medium-high) pulmonary TB, aged ≥10 years were eligible for recruitment; some HHC were recruited to the ERASE-TB cohort outside the recruitment window for the current study and thus were ineligible for this study [15].

Among 314 people with TB, 229 were able to be contacted at the time of or after completing TB treatment, of the remainder 20 (6.4% of the total) had died and 65 (20.7%) could not be reached (Fig 1). Of those contacted, 96 (42%) participated. Common reasons for non-participation were having permanently or temporarily moved away (often for work) or being busy with work and thus unable to attend. Among participants, the median interval from TB diagnosis was 356 days (interquartile range 215–488 days; Table D in S1 Appendix). Males were less likely to participate; whilst distributions of age, employment and HIV status were similar between those who did and did not participate (Table E in S1 Appendix). Among people with recent TB, completion of all screening components was high, other than for impaired lung function where only 57/96 (59%) completed spirometry (Figure A in S1 Appendix). Of those without spirometry, 19 had contraindications or were considered too unwell to perform spirometry by the technician, whilst for 19 spirometry was not done but no reason for missingness was recorded. Only one person refused spirometry. For the following analyses, 92 people who completed all screening components other than spirometry are included; analyses of impaired lung function are in the subgroup with good quality post-bronchodilator spirometry available (n = 39/92). Comparison of people with TB with valid spirometry to those who did not identified that people without spirometry were more likely to have cough (49% vs 7.7%; p < 0.001), and may be older (median age 38 vs 35 years; p = 0.10) and more often female (53% vs 36%; p = 0.11; Table F in S1 Appendix).

#### Household comparators without TB.

From 467 members of TB-affected households who participated, 285 were aged 18 years or older, did not have a history of previous TB, were not diagnosed with TB in the study and had complete data on screening tests other than spirometry, and thus were included as comparators for this analysis. Spirometry data were available for 155/285 included household comparators. Of the included comparators, 112 were from the same households as the participants with recent TB. Of 192 households included in this study, 87% were living in poverty, 27% were crowded and 12% used biomass fuels (Table G in S1 Appendix).

#### Characteristics of people with recent TB and household comparators.

People with recent TB were older than the household comparators but had a narrower age range (median 38 [IQR 28–44] vs 34 [24–46] years; Figure B in S1 Appendix). People with recent TB were more likely to be men (n = 50/92, 54% vs n = 84/285, 29%; Table 1). Cough was common among both people with TB and household comparators (n = 29/92, 32% vs n = 42/285, 15%; p = 0.001) while other TB related symptoms were uncommon (Tables H and I in S1 Appendix). Although 73% (n = 67) of people with recent TB rated their health as better than at the time of TB diagnosis, 27% (n = 25) reported that their health was the same or worse (Table J in S1 Appendix).

**Table 1:** Characteristics of people with recent TB and household comparators, with age and sex-adjusted p values.

Characteristic		Household comparators N = 285	People with recent TB N = 92	p value*
**Demographics**				
**Gender (n=377)**	Men	84 (29%)	50 (54%)	–
	Women	201 (71%)	42 (46%)	–
**Age, years**		34 (24–46)	38 (28–44)	–
**School attainment (n=356)**	At least secondary	230 (85%)	73 (85%)	0.39
**Medical aid cover (n=375)**		25 (8.8%)	5 (5.5%)	0.22
**Currently pregnant (n=221)†**		17 (9.2%)	2 (5.4%)	0.48
**History of work in mining (n=353)**		13 (4.6%)	11 (16%)	0.09
**History of migration to South Africa (n=353)**		48 (17%)	13 (19%)	0.95
**History of imprisonment (n=353)**		8 (2.8%)	7 (10%)	0.22
**Health behaviours**				
**Current/former tobacco smoker (n=377)**		40 (14%)	25 (27%)	0.61
**Alcohol use category (AUDIT) (n=377)§**	Non-drinker	194 (68%)	57 (62%)	0.57
	Non-harmful drinking	58 (20%)	21 (23%)	
	Harmful/dependent	33 (12%)	14 (15%)	
**AUDIT score§**		5 (2–11)	4 (3–14)	0.24
**Use of other substances (n=366)**¶		40 (14%)	23 (26%)	0.26
**≥1 COVID-19 vaccination received (n=377)**		161 (56%)	51 (55%)	0.75
**Symptoms**				
**Cough (n=377)**		42 (15%)	29 (32%)	0.001
Duration (weeks) ||		1.00 (0.54–2.00)	4.35 (1.00–21.7)	0.033
**TB symptom screen positive (n=377)**		45 (16%)	30 (33%)	0.002
Xpert positive (n=75)**		0 (0.0%)	6 (35%)	–

Presented as number (percentage) or median (interquartile range). Wald p values from logistic regression models, adjusted for age (4-level categorical variable: 18–29, 30–39, 40–49 and 50 + years) and sex, with TB status as the outcome presented. 95% confidence intervals (95%CI) use robust standard errors to account for household clustering. * Education status missing for 20 people; history of wrok in mining, migration to South Africa and imprisonment missing for 24 people with recent TB. † Pregnancy reported among women only. § Alcohol use disorders identification test (AUDIT) score categorised as harmful/dependent if score ≥8; quantitative scores are presented among people who reported drinking any alcohol (i.e., ‘non-drinkers’ are excluded from the denominator). ¶ Use of substances measured using the alcohol, smoking and substance involvement screening test (ASSIST) tool, with any participant responding yes to use of any of cannabis, cocaine, amphetamine-type stimulants, sedatives and sleeping pills, hallucinogens, inhalants, opioids or other drugs categorised as ‘yes’. 4 people with TB and 7 household comparators who responded ‘don’t know’ to use of substances are not shown in the table. Of 63 people who disclosed drug use, 29 (46%) reported use of cannabis, 5 reported use of methamphetamines and 2 people reported using cocaine. || Duration of cough is among people reporting cough only. ** Xpert MTB/Rif Ultra performed if positive symptom screen; 13 Xpert missing among people with TB. Of the people with recent TB, all results were ≤ semi-quantitative low. All positive or unknown results were followed up with clinical review and repeat Xpert, none were confirmed TB. Among household comparators, people diagnosed with TB in the study were explicitly excluded from this analysis.

#### Chronic conditions among people with TB and household comparators.

**Fig 2:**
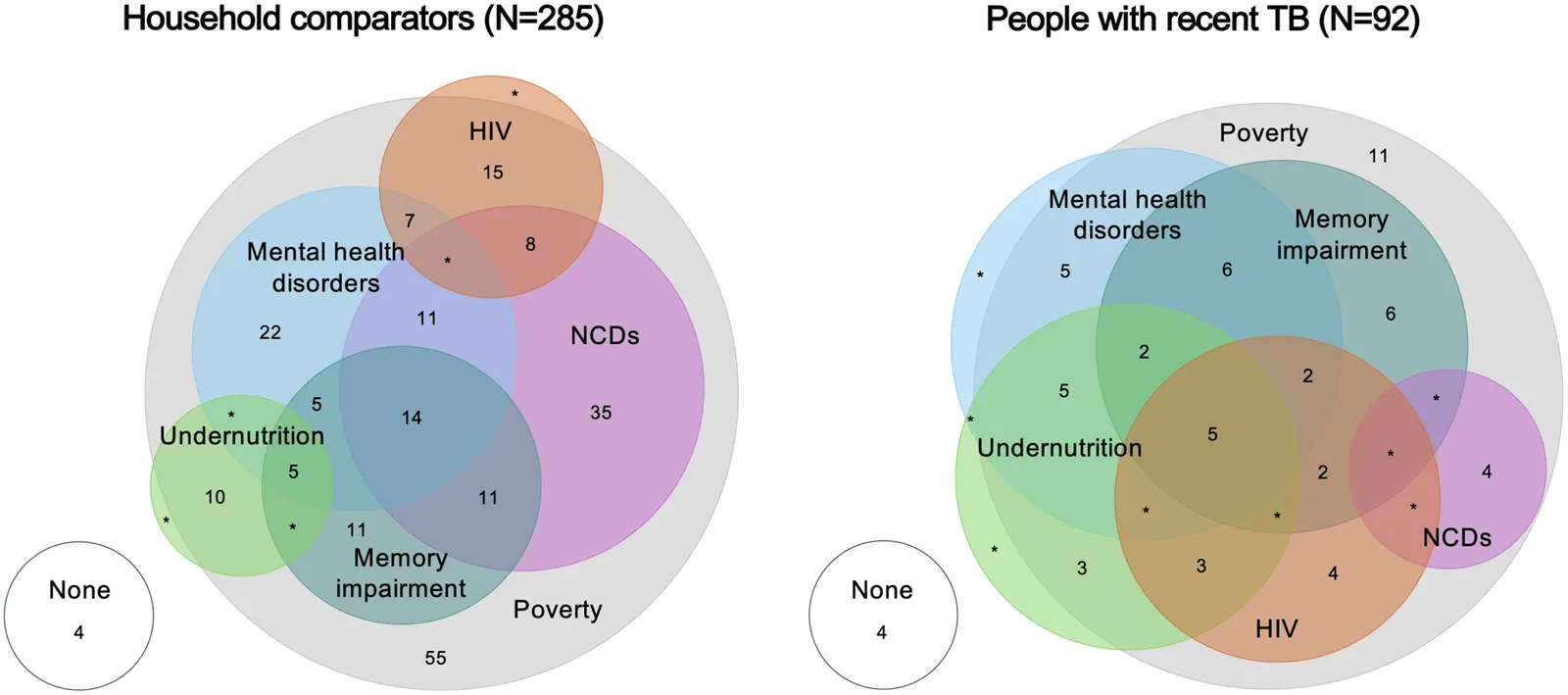
Euler plot illustrating the overlap of chronic conditions among people with recent TB and household comparators, superimposed on household poverty. Poverty is defined as household income <2.15 United States Dollars per person per day and is measured at household level. All other variables are measured at individual level. Non-communicable diseases (NCDs) defined as either diabetes or hypertension. Undernutrition defined as either underweight (suggestive of macronutrient deficiency) or anaemia (suggestive of micronutrient deficiency). Lung function impairment and vision impairment are excluded from the figure. Observed (unadjusted) numbers are presented; ‘None’ represents none of the five plotted groupings, * indicates intersections with ≤1 person.

The prevalence of chronic conditions was high in both people with TB and household comparators, with 91% of people with TB (n = 84/92) and 81% (n = 231/285) of household comparators having at least one condition (Table 2; Table K in S1 Appendix). People with recent TB were more likely to have at least one condition (aOR 2.30; 95%CI 1.03–5.11) compared to household comparators after adjustment for age and sex, but the association with multimorbidity was not statistically significant (two or more conditions: n = 57/92 (62%) people with recent TB vs n = 154/285 (54%) household comparators; aOR 1.55, 95%CI 0.86–2.78). The relative contribution of different conditions to multimorbidity was different between groups (Fig 2). In a sensitivity analysis excluding self-reported memory impairment as a component of multimorbidity the corresponding estimates were smaller (at least one condition: n = 80/92 (87%) vs n = 223/285 (78%), aOR 1.70 [0.83–3.49]; multimorbidity: n = 47 (51%) vs n = 137 (48%), aOR 1.18 [0.68–2.06]; Table L in S1 Appendix).

**Table 2:** Odds ratios for chronic conditions, among people with recent TB versus household comparators, after accounting for age and sex.

Characteristic	Household comparators (N = 285)	People with recent TB (N = 92)	aOR*	p	aOR + HIV*	p
**Multimorbidity**						
**≥1 chronic condition**	231 (81%)	84 (91%)	2.30 (1.03, 5.11)	0.04	–	
**Multimorbidity**†	154 (54%)	57 (62%)	1.55 (0.86, 2.78)	0.14	–	
**Infectious diseases**						
**HIV**	61 (21%)	28 (31%)	1.77 (1.03, 3.04)	0.04	–	
**Non-communicable diseases**						
**Diabetes**‡	25 (8.8%)	11 (12%)	1.61 (0.75, 3.44)	0.22	1.59 (0.74, 3.39)	0.23
**Hypertension**	98 (34%)	18 (20%)	0.40 (0.20, 0.79)	0.009	0.45 (0.22, 0.88)	0.02
**Self-reported memory difficulty**	69 (24%)	35 (38%)	2.13 (1.19, 3.80)	0.01	2.00 (1.11, 3.39)	0.02
**Symptoms of mental health disorders**	98 (34%)	41 (45%)	1.57 (0.96, 2.56)	0.07	1.53 (0.93, 2.52)	0.10
**Vision impairment**	89 (31%)	25 (27%)	0.71 (0.37, 1.35)	0.29	0.68 (0.35, 1.32)	0.25
**Impaired lung function**‡¶	33 (21%)	18 (46%)	3.24 (1.46, 7.20)	0.004	3.43 (1.54, 7.64)	0.003
**Nutritional disorders**						
**Underweight**‡	14 (4.9%)	22 (24%)	5.15 (2.28, 11.61)	<0.001	5.30 (2.36, 11.90)	<0.001
**Anaemia**‡	27 (9.5%)	9 (9.8%)	1.23 (0.56, 2.73)	0.60	1.10 (0.52, 2.36)	0.80
**Quality of life and physical activity**						
EQ-5D value**	0.90 (0.85, 0.90)	0.86 (0.84, 0.90)	-0.03 (-0.04, -0.01)	0.005	–	
Physical activity††	3580 (1483, 7521)	4800 (1271, 9586)	-532 (-2280, 1217)	0.55	–	

Outcomes presented and number (percentage) or median (interquartile range, for continuous variables). * aOR are odds ratios (and 95% confidence intervals) adjusted for age (4-level categorical variable: 18–29, 30–39, 40–49 and 50 + years) and sex; aOR + HIV are additionally adjusted for HIV status using logistic regression. All 95% confidence intervals are adjusted for household level clustering using robust standard errors. P values are from Wald tests. † Multimorbidity defined as ≥2 of HIV, diabetes, hypertension, self-reported memory difficulty, symptoms of mental health disorders, vision impairment, impaired lung function, underweight or anaemia. ‡ In models for indicated outcomes, a linear term was used to adjust for age to avoid over-parameterisation of the model. ¶ Impaired lung function is calculated among people with good quality (A-C grade) post-bronchodilator spirometry only (n = 155/285 household comparators and n = 39/92 people with recent TB) and defined as any of obstructive, restrictive or mixed defects. ** EQ-5D value as a continuous outcome; 2 people were missing EQ-5D data. †† Metabolic equivalent task-minutes [MET-min] per week. Abbreviations: TB = tuberculosis.

Regarding individual conditions, HIV, underweight, symptoms of mental health disorders, self-reported memory difficulty and impaired lung function were more common among people with recent TB than comparators after adjustment for age and sex (HIV: n = 28/92 (31%) vs n = 61/285 (21%); aOR 1.77 [95%CI 1.03–3.04]; underweight: n = 22/92 (24%) vs n = 14/285 (4.9%), aOR 5.15 [2.28–11.61]; symptoms of mental health disorders: n = 41/92 (45%) vs n = 98/285 (34%), aOR 1.57 [0.96–2.56]; self-reported memory difficulty: n = 35/92 (38%) vs n = 69/285 (24%), aOR 2.13 [1.19–3.80]; impaired lung function: n = 18/39 (46%) vs n = 33/155 (21%), aOR 3.24 [1.46–7.20]). Most lung function impairment, in both groups, was a restrictive pattern (n = 17/18 people with recent TB and impaired spirometry and 30/33 household comparators). Impaired lung function was more common, and the association with recent TB stronger when the GLI Global equation was used as the basis of z-score calculations (n = 27/39 people with recent TB (69%) vs n = 49/155 comparators (32%); aOR 4.71 (2.11–10.49, p < 0.001). Self-reported memory difficulty was positively associated with age and symptoms of mental health disorders (Table M and Table N in S1 Appendix); however among participants who screened negative on SSQ, the direction of association persisted (n = 16/51 people with recent TB vs n = 31/187 household comparators; aOR 2.64, 95%CI 1.17–5.94; p = 0.019; Table O in S1 Appendix). Point estimates suggested higher odds of diabetes among people with recent TB versus household comparators after adjustment for age and sex (aOR 1.61 [0.75–3.44]), albeit without statistical evidence for a difference (p = 0.2; Table 2). Odds of hypertension were lower among people with recent TB (aOR 0.40 [0.20–0.79]). These associations persisted after adjustment for HIV status and were partially attenuated after adjustment for BMI (Table P in S1 Appendix). Table 3 shows the predicted proportion of people with recent TB and household comparators with each chronic condition, after adjustment for age and sex.

**Table 3:** Predicted proportion of people with each chronic condition after adjustment for age and sex.

	Household comparators N = 285	People with recent TB N = 92
**≥1 chronic condition**	86.2% (80.8–91.5%)	93.5% (87.8–99.2%)
Multimorbidity*	54.4% (46.7–62.0%)	64.8% (53.1–76.5%)
**HIV**	18.8% (13.1–24.4%)	29.1% (19.5–38.7%)
**Diabetes**	7.1% (3.9–10.3%)	11.0% (4.8–17.2%)
**Hypertension**	29.8% (22.6–37.1%)	14.5% (6.9–22.0%)
**Self-reported memory difficulty**	22.0% (16.3–27.7%)	37.5% (27.2–47.8%)
**Symptoms of mental health disorders**	33.9% (27.9–39.9%)	44.6% (34.2–54.9%)
**Vision impairment**	27.2% (20.2–34.2%)	20.9% (11.4–30.3%)
**Impaired lung function**	21.1% (14.2–28.0%)	46.4% (30.5–62.3%)
**Underweight**	4.6% (1.6–7.6%)	19.9% (10.7–29.1%)
**Anaemia**	7.9% (4.6–11.1%)	9.6% (3.2–16.0%)

Predicted proportions estimated using margins command, at the mean age of people with TB (30–39 years) and assuming equal numbers of men and women in the population.

No people with recent TB tested newly positive for HIV, whilst four household comparators did so. In contrast, other chronic diseases were undiagnosed among at least half of people (49%–100% of prevalent disease undiagnosed), with no evidence that conditions among people with TB were more likely to have been diagnosed compared to people who had not recently been engaged in TB care (Table Q in S1 Appendix).

Stratification by sex suggested effect modification after adjustment for age, although these analyses should be considered exploratory and confidence intervals overlapped throughout (Table R in S1 Appendix). The association between recent TB and having at least one chronic condition or multimorbidity was stronger in men compared to women (aOR 4.13 [1.08–15.79] vs 1.55 [0.53–4.56] and 2.39 [1.09–5.22] vs 1.01 [0.44–2.32]). For HIV (aOR 3.23 [1.31–7.92] among men vs 1.21 [0.56–2.61] among women), impaired lung function (4.62 [1.36–15.71] vs 2.71 [0.83–8.92]) and symptoms of mental health disorders (2.10 [1.00–4.42] vs 1.18 [0.58–2.41]), the association between recent TB and chronic diseases was stronger among men; whilst for self-reported memory difficulties it was stronger among women (aOR 2.68 ([1.29–5.55] vs 1.63 [0.69–3.90]). The association between recent TB and underweight was evident in both groups in stratified analyses, but point estimates were larger in women (aOR 6.82 [1.72–27.05] in women vs 4.70 [1.60–13.79] in men).

#### Health-related quality of life and physical activity.

The mean EQ-5D value in the overall study population was 0.86 (SD 0.072) thus the MCID was defined as 0.036. HRQoL was lower among people with TB compared to household comparators (median EQ-5D value 0.86 (interquartile range 0.84–0.90) vs 0.90 (0.85–0.90), p = 0.007). Over half of people with TB reported having at least some difficulty in one or more of the EQ-5D domains (Table S in S1 Appendix). After adjustment for age and sex, people with recent TB were more likely to have lower HRQoL than household comparators (regression coefficient -0.03 (-0.04, -0.01), p = 0.005, Table 2), but this did not exceed the MCID. In a sensitivity analysis with ordinal logistic regression, the same association was observed (Table T in S1 Appendix).

Self-reported physical activity using IPAQ-SF was high in both groups; there was no evidence for lower physical activity among people with recent TB.

## Discussion

This study demonstrated a high prevalence of chronic conditions among people with TB, with evidence that HIV, underweight, impaired lung function and self-reported memory difficulty were more common among people with a recent history of TB than household comparators. The point estimate for diabetes also suggested higher odds among people with recent TB, but with no statistical evidence for a difference; this may reflect limited power to detect associations for a relatively uncommon condition. Assessed an average of a year after TB diagnosis, a quarter of people were still underweight and almost half (45%) had symptoms of mental health disorders. It is striking that, despite people with TB having been regularly engaged in health services for six months of TB treatment, these conditions were mostly unrecognised, and people with TB were no more likely to have their conditions identified previously than household comparators. Notably, no new diagnoses of HIV were made among people with recent TB in this study. This reflects the impact of a strong national HIV programme including well-embedded HIV-TB integration, however it should be noted that one third of the people with TB in this study reported being diagnosed with HIV and TB at the same time [28]. Overall, almost two-thirds of people with recent TB had multimorbidity (≥2 conditions) after TB treatment completion – if TB itself is considered a morbidity (≥1 condition and TB, as has been defined previously [9]), nine in ten did so.

Together, these findings suggest far-reaching consequences of pulmonary TB beyond respiratory impairment among TB survivors, and underscore the critical need to implement integrated non-TB health services for people with TB. Integration of non-TB services into TB treatment platforms may be an important opportunity to reach a population largely living in poverty with a high burden of chronic conditions and who may not otherwise access care [29]. At a public health level, an estimated 58 million disability-adjusted life years are attributable to post-TB sequelae [6]. However, these models are based solely on estimates of chronic respiratory disease among TB survivors. The findings of this study, together with other emerging evidence, suggest that chronic respiratory disease represents only part of the sequelae of TB – and consequently that the overall morbidity and mortality attributable to TB may be substantially higher than currently estimated.

Our findings on post-TB lung impairment are broadly consistent with previous reports; a smaller effect size compared with other studies [10] may reflect better matching of comparators for socio-economic and environmental determinants of lung health at household or community level, or choice of reference equations for interpretation; however this should also be interpreted with caution given the small sample size and consequent wide confidence intervals. Data on other chronic conditions among people with recent TB are limited. A population registry data-based study in Canada [30] reported higher odds of multimorbidity (OR 1.74) and distinct multimorbidity patterns among people with previous TB compared to those without, whilst registry-based analyses in high-income settings have reported excess cardiovascular morbidity and mortality among people with previous TB compared to people without prior TB [31]. Consistent with our findings, a study from Peru found that people with a history of recent TB were less likely to be hypertensive (prevalence ratio 0.31 [95%CI 0.10–0.98]) and more likely to be diabetic (assessed using glycosuria; 3.66 [1.68–8.01]) than randomly selected community members [14]. Several mechanisms may contribute to this lower prevalence of hypertension. Here, adjustment for BMI partially attenuated the association: TB-associated underweight may lower blood pressure through reduced vascular resistance and sodium depletion. In addition, individuals with hypertension may experience higher early mortality during TB treatment as a result of increased risk of precipitated cardiovascular events, and thus be under-represented in a post-treatment cohort [31]. Unpicking the association between TB and long-term cardiovascular health requires further work: TB is associated with cardiac complications during acute disease (such as pericarditis and pericardial effusions) and post-TB (pulmonary hypertension) therefore it is possible that the mechanisms of post-TB cardiac disease differ from those in the general population and are not fully captured by common cardiovascular risk assessment tools [13,32]. Nevertheless, measurement of blood pressure is simple and quick and hypertension is highly prevalent and is a major risk factor for cardiovascular disease, the leading cause of death globally. As a result, inclusion of blood pressure measurements as part of post-TB assessments remains appropriate [33]. The excess risk of diabetes, HIV, mental health and undernutrition among TB survivors in our study reiterates the urgent need for implementation of WHO guidance endorsing screening for these conditions at TB diagnosis, and during treatment [34].

Whilst the need to better understand neurocognitive impacts of central nervous system TB is increasingly recognised [13], our finding that 38% of people with recent pulmonary TB self-reported memory difficulty (using a very brief screening tool) was unexpected. Reported memory difficulty among household comparators without TB was also high (24%), perhaps suggesting a lack of specificity in the tool. Memory difficulty is generally associated with older age as dementia is more common in older people; the association between older age and increased prevalence of memory difficulty observed here supports construct validity and biological plausibility. Self-reported memory difficulty was strongly associated with symptoms of mental health disorder; thus the former may reflect cognitive dysfunction due to affective disorders (e.g., difficulty with concentration). However the association did persist among the subset of the population who screened negative for mental health disorders. The only previous data on neurocognitive impacts of pulmonary TB are,to our knowledge, from one small study in South Africa which investigated neurocognitive function among people with CNS TB compared to control groups [35]. We used a quick single question screening tool, developed in a high-income setting and not yet validated locally, hence measurement error cannot be excluded. As such, this unexpected finding requires validation through further study and consideration of possible underlying mechanisms.

Participation in the post-treatment health check among people with TB was low. This is consistent with previous studies from Peru and five African sites in which 35% and 52% of people initiated on TB treatment participated in a post-TB assessment respectively, but is lower than that found in a study from Uganda, Zambia and Zimbabwe that integrated end-of-treatment screening into routine clinic procedures, and where 78% of people identified at end of treatment were assessed [5,12,14]. The reasons for low uptake among people with TB are likely multifactorial and may include competing priorities such as the need to generate income in the context of profound financial insecurity, and relatively low trust in healthcare services. Discussion with our local TB community advisory board highlighted additional contextual factors in Zimbabwe: lower accessibility and negative perceptions of rural health services in Zimbabwe, together with the preference for family-based care during illness, often lead people to seek care in urban centres before returning to other areas once they have recovered. Economic opportunity is also a major driver of internal and external migration in this setting. The low uptake of the integrated health check suggests post-TB clinical evaluations delivered as a stand-alone intervention after TB treatment completion are unlikely to be effective. Offering services earlier, during TB treatment, and integrating these into the routine mechanisms of TB care delivery may reduce the opportunity cost of participation. At the same time person-centred approaches to care help build trust in health services and support healthier behaviours wirth benefits which could last well beyond the period of engagement in TB care.

Strengths of this study include measurement of all the domains recommended for evaluation of post-TB conditions, and more [13]. We provide the first evidence from Africa on the burden of multimorbidity among people with recent TB, in comparison to unexposed comparators, addressing a key priority identified by post-TB symposium experts by determining how much post-TB morbidity may be attributable to TB itself rather than pre-existing comorbidities. Our comparators were selected from TB-affected households ensuring close matching between people with TB and those without with respect to social and structural determinants of poor health; conversely however the economic and caregiving burden of TB on household members may have impacted comparators’ health, particularly their HRQoL and mental health, attenuating the between-group difference through externality. In addition, we objectively assessed for chronic conditions, overcoming the risk of under detection due to lack of access to diagnosis when using self-report.

Limitations of the study include that, as per the enrolment criteria of the ERASE-TB study, this study only included people with TB who had medium-high grade positive Xpert MTB/Rif Ultra at diagnosis. As the degree of Xpert positivity is related to TB disease severity, the resultant exclusion of individuals with lower bacterial burden means that people with milder forms of the disease (who may have lower prevalence of associated conditions and complications) are not represented here. Secondly, as above, about 40% of the identified people with TB participated in the study, it is possible that those who chose to participate had more health concerns than those who did not. Participation bias therefore may have resulted in over-estimation of chronic condition prevalence in the TB group. Given high participation in the integrated health check among household contacts, differential participation may also have led to over-estimation of the association between recent TB and chronic conditions. Incomplete participation by people with recent TB also resulted in a small sample size and thus uncertainty in effect estimates. In particular, there were substantial missing data for spirometry; of those missing this data, half were considered too unwell by the research nurse; these people may have been those with the greatest respiratory impairment resulting in under-estimation of respiratory disease in this study; our assumption that those with missing lung function data did not have impairment in calculation of multimorbidity means the prevalence reported is likely to be an under-estimate. In other cases, reason for missingness was not recorded. Whilst several health conditions were included, there were notable exceptions: we did not look at disability or functional assessments (such as the 6-minute walk test), or evaluate stigma and social exclusion [5,13]. Household comparators were also enrolled approximately one year earlier than people with recent TB, and period effects, such as economic shocks or the sequelae of COVID-19, may have differentially affected the two groups. The magnitude of associations between recent TB status and health-related quality of life was relatively small and may not be clinically significant. Utility values in this study were derived by crosswalk from EQ-5D-5L to the Zimbabwe EQ-5D-3L value set since no EQ-5D-5L value set exists for Zimbabwe; in other settings crosswalk-derived utilities differ from those directly elicited and can be compressed in the upper utility range [36]. Consequent measurement uncertainty could contribute to the modest between-group difference observed. IPAQ-SF can over-estimate physical activity, particularly for moderate and incidental activity and performs less well in low-income populations, where manual labour predominates, compared to the high-income settings in which it was derived; the lack of association here should be interpreted with caution [37]. We assessed chronic conditions at a median of one year after TB diagnosis: studies with longer follow up are needed to ascertain the longer term course of post-TB multimorbidity. Finally, despite robust selection of household comparators, several included chronic conditions may be causes of TB, consequences of the disease, or both – causality cannot be inferred from these analyses. As in the post-TB lung disease literature more widely, whilst our findings suggest multimorbidity among people with recent TB is attributable at least in part to previous tuberculosis disease [38], the degree to which this reflects the consequences of as opposed to pre-existing disease, is unclear.

In conclusion, this study demonstrated high prevalence of chronic conditions and multimorbidity among people with recent TB, with worse HRQoL compared to people from the same households who have never had TB. This warrants further evaluation in future studies, including longer follow up, whilst the contribution of non-respiratory multimorbidity to the total disability adjusted life years attributable to TB should be considered in modelling estimates. Importantly, however, participation in our integrated health check by people with recent TB was relatively low. There is an urgent need to develop not just feasible, but desirable, interventions to identify and address chronic conditions among people with TB, ensuring that these have low opportunity cost and fit alongside other urgent and fundamental priorities. It is essential that these interventions are designed with community buy-in and contextual understanding. Addressing post-TB (multi)morbidity is likely to yield important benefits not only for individuals (e.g., through reduced mortality, reduced disability, and improved quality of life) but also for families (e.g., care burden, financial security) and societies, by retaining an economically critical workforce [1,3].

## Supporting information

S1 Appendix Supplementary methods and results. (DOCX)
