# Supplementary material for: Multimorbidity and health-related quality of life after tuberculosis treatment in Zimbabwe: A cross-sectional study with household comparators
Source: PLOS Glob Public Health. 2026 Sep 1;6(9):e0007119. doi: 10.1371/journal.pgph.0007119 (PMC13533362; doi:10.1371/journal.pgph.0007119)
Supplement: Supplementary file 1 [file pgph.0007119.s001.docx]

**Multimorbidity and health-related quality of life after tuberculosis treatment in Zimbabwe: a cross-sectional study with household comparators**

## S1 APPENDIX

## Table A: Completed STROBE checklist

|  | **Item No.** | **Recommendation** | **Line No.** |
| --- | --- | --- | --- |
| **Title and abstract** | 1 | (a) Indicate the study’s design with a commonly used term in the title or the abstract | 1 |
|  |  | (b) Provide in the abstract an informative and balanced summary of what was done and what was found | 37 |
| **Introduction** | | | |
| **Background/ rationale** | 2 | Explain the scientific background and rationale for the investigation being reported | 71 |
| **Objectives** | 3 | State specific objectives, including any prespecified hypotheses | 95 |
| **Methods** | | | |
| **Study design** | 4 | Present key elements of study design early in the paper | 107 |
| **Setting** | 5 | Describe the setting, locations, and relevant dates, including periods of recruitment, exposure, follow-up, and data collection | 107 |
| **Participants** | 6 | (a) Cohort study–Give the eligibility criteria, and the sources and methods of selection of participants. Describe methods of follow-up  Case-control study–Give the eligibility criteria, and the sources and methods of case ascertainment and control selection. Give the rationale for the choice of cases and controls  Cross-sectional study–Give the eligibility criteria, and the sources and methods of selection of participants | 115 |
|  |  | (b) Cohort study–For matched studies, give matching criteria and number of exposed and unexposed  Case-control study–For matched studies, give matching criteria and the number of controls per case | NA |
| **Variables** | 7 | Clearly define all outcomes, exposures, predictors, potential confounders, and effect modifiers. Give diagnostic criteria, if applicable | 142 |
| **Data sources/ measurement** | 8* | For each variable of interest, give sources of data and details of methods of assessment (measurement). Describe comparability of assessment methods if there is more than one group | 120 |
| **Bias** | 9 | Describe any efforts to address potential sources of bias | 160 |
| **Study size** | 10 | Explain how the study size was arrived at | 183 |
| **Quantitative variables** | 11 | Explain how quantitative variables were handled in the analyses. If applicable, describe which groupings were chosen and why | 160 |
| **Statistical methods** | 12 | (a) Describe all statistical methods, including those used to control for confounding | 160 |
|  |  | (b) Describe any methods used to examine subgroups and interactions | 172 |
|  |  | (c) Explain how missing data were addressed | 138 |
|  |  | (d) Cohort study–If applicable, explain how loss to follow-up was addressed  Case-control study–If applicable, explain how matching of cases and controls was addressed  Cross-sectional study–If applicable, describe analytical methods taking account of sampling strategy | NA |
|  |  | (e) Describe any sensitivity analyses | 150 |
| **Results** | | | |
| **Participants** | 13* | (a) Report numbers of individuals at each stage of study–eg numbers potentially eligible, examined for eligibility, confirmed eligible, included in the study, completing follow-up, and analysed | 199 |
|  |  | (b) Give reasons for non-participation at each stage | 200 |
|  |  | (c) Consider use of a flow diagram | 200 |
| **Descriptive data** | 14* | (a) Give characteristics of study participants (eg demographic, clinical, social) and information on exposures and potential confounders | 202 |
|  |  | (b) Indicate number of participants with missing data for each variable of interest | 210 |
|  |  | (c) Cohort study–Summarise follow-up time (eg, average and total amount) | NA |
| **Outcome data** | 15* | Cohort study–Report numbers of outcome events or summary measures over time | NA |
|  |  | Case-control study–Report numbers in each exposure category, or summary measures of exposure | 240 |
|  |  | Cross-sectional study–Report numbers of outcome events or summary measures | 237 |
| **Main results** | 16 | (a) Give unadjusted estimates and, if applicable, confounder-adjusted estimates and their precision (eg, 95% confidence interval). Make clear which confounders were adjusted for and why they were included | 273 |
|  |  | (b) Report category boundaries when continuous variables were categorised | 237 |
|  |  | (c) If relevant, consider translating estimates of relative risk into absolute risk for a meaningful time period | 281 |
| **Other analyses** | 17 | Report other analyses done–eg analyses of subgroups and interactions, and sensitivity analyses | 288 |
| **Discussion** | | | |
| **Key results** | 18 | Summarise key results with reference to study objectives | 294 |
| **Limitations** | 19 | Discuss limitations of the study, taking into account sources of potential bias or imprecision. Discuss both direction and magnitude of any potential bias | 410 |
| **Interpretation** | 20 | Give a cautious overall interpretation of results considering objectives, limitations, multiplicity of analyses, results from similar studies, and other relevant evidence | 311 |
| **Generalisability** | 21 | Discuss the generalisability (external validity) of the study results | 381 |
| **Other information** | | | |
| **Funding** | 22 | Give the source of funding and the role of the funders for the present study and, if applicable, for the original study on which the present article is based | 483 |

## Table B: Screening procedures for chronic conditions, health-related quality of life and physical activity, and definitions used in the study

| **Condition** | **Screening test(s)** | **Definition of condition** |
| --- | --- | --- |
| HIV | HIV 1/2 blood based rapid test positive (as per national guidelines) | Positive screening and confirmatory test |
| Underweight | Measurement of height (fixed stadiometer) and weight (digital scale; Seca) to calculate body mass index (BMI) | BMI <18.5kg/m^2^ |
| Vision impairment | Peek Acuity app for distance vision and, additionally for participants aged ≥40 years, near vision | Score 6/12 or worse for distance and/or near vision on either eye |
| Anaemia | Haemoglobin measured using Hemocue 301 (Hemocue) | WHO age and sex-stratified definitions of anaemia^1^ |
| Diabetes | HbA1c measured using A1c Care (SD Biosensor) | HbA1c ≥6.5% |
| Hypertension | Blood pressure measured twice 5 minutes apart, and a third time if either elevated, as per WHO STEPs protocol, and repeated a third time if either BP was elevated.^1^ | Second BP ≥140/90mmHg, or average of second and third BP ≥140/90mmHg if a third BP performed. |
| Mental health | Shona Symptom Questionnaire in ACASI format^2^ | SSQ ≥8 or red flags present (red flags are defined as answering yes to suicidal thoughts or hallucinations) |
| Alcohol use disorder | Alcohol Use Disorder Identification Test (AUDIT) in ACASI format^3^ | Score ≥8 |
| Substance use | Alcohol, Smoking and Substance Involvement Screening Test (ASSIST) in ACASI format^4^ | Any participant responding yes to use of any of cannabis, cocaine, amphetamine-type stimulants, sedatives and sleeping pills, hallucinogens, inhalants, opioids or other drugs. |
| Impaired lung function | Pre-and post-bronchodilator spirometry (Easy-on-PC, EasyOne), performed according to ERS/ATS technical standards^5^, including external quality assurance and grading. | Impaired lung function (obstructive, restrictive or mixed pattern), as defined in ERS/ATS standards^6^ and using the GLI African American reference equation. |
| Memory impairment | ‘In the past 12 months, have you had difficulty remembering things or following a story or conversation?’ | ‘Yes’ answer |
| Health-related quality of life | EQ-5D-5L | Values calculated using crosswalk to Zimbabwe value set using EuroQOL methods.^7^ |
| Physical activity | International Physical Activity Questionnaire-Short Form | High physical activity defined as either a) vigorous-intensity activity on at least 3 days and accumulating at least 1500 MET-minutes/week or b) 7 days of any combination of walking, moderate-intensity or vigorous intensity activities achieving a minimum of at least 3000 MET-minutes/week.^8^ |

## Table C: Illustrative power of the study sample size to detect odds ratios of varying magnitude, at the 95% confidence level

| **Outcome** | **N (TB vs comparators)** | **Comparator prevalence** | **OR 1.5** | **OR 2.0** | **OR 2.5** | **OR 3.0** |
| --- | --- | --- | --- | --- | --- | --- |
| ≥1 chronic condition | 92 vs 285 | 81% | 21% | 47% | 68% | **81%** |
| Multimorbidity | 92 vs 285 | 54% | 37% | **79%** | **95%** | **99%** |
| HIV | 92 vs 285 | 21% | 33% | **74%** | **94%** | **99%** |
| Underweight | 92 vs 285 | 5% | 15% | 37% | 58% | 75% |
| Impaired lung function | 39 vs 155 | 21% | 18% | 44% | 67% | **83%** |

## Table D: TB treatment outcome among participants with recent TB

|  |  | N (%) | Median interval from TB diagnosis in days (IQR) |
| --- | --- | --- | --- |
| **Overall** |  | 96 (100%) | 356 (215–488) |
| **TB treatment status*** | Completed TB treatment | 71 (73.9%) | 379 (324–524) |
|  | Currently taking TB treatment | 20 (20.8%) | 164.5 (154–175) |
|  | Lost to follow-up from TB treatment | 3 (3.1%) | 592 (424–614) |
|  | Missing | 2 (2.1%) | 366 (365–367) |
| **Footnotes**: * as reported by the participant. Reported as median (interquartile range) or number (percentage). Missing means that the TB treatment status was not recorded. | | | |

## Table E: Comparison of characteristics of people with recent TB who participated in the intervention to those who did not (N=314)

| Characteristic |  | Did not participate  N = 218 | Participated  N = 96 | p-value |
| --- | --- | --- | --- | --- |
| **Age at baseline, years** | | 36 (27–42) | 37 (27–43) | 0.4 |
| **Female** |  | 63 (29%) | 42 (44%) | 0.01 |
| **Employment** | Formal | 19 (8.7%) | 4 (4.2%) | 0.4 |
|  | Informal | 74 (34%) | 37 (39%) |  |
|  | Unable to work | 95 (44%) | 39 (41%) |  |
|  | Unemployed | 22 (10%) | 9 (9.4%) |  |
|  | Other | 8 (3.7%) | 7 (7.3%) |  |
| **HIV status** | Unknown | 14 (6.4%) | 6 (6.3%) | 0.3 |
|  | Negative | 123 (56%) | 64 (67%) |  |
|  | Positive, on ART | 51 (23%) | 14 (15%) |  |
|  | Positive, not on ART | 30 (14%) | 12 (13%) |  |
| **Footnotes**: Reported as median (interquartile range) or number (percentage). P values from Wilcoxon rank sum test or Pearson’s Chi-squared test. | | | | |

## Figure A: Uptake of individual screening components

**Footnotes:** Of those without spirometry, 19 had contraindications or were considered too unwell to perform spirometry by the technician, whilst for 18 spirometry was not done but no reason for missingness was recorded. Only one person refused spirometry.

## Table F: Characteristics of people with recent TB, by availability of good-quality spirometry

| **Characteristic** | **Valid spirometry** | **No valid spirometry** | **p value*** |
| --- | --- | --- | --- |

| **People with recent TB** | **N = 39^1^** | **N = 53** |  |
| --- | --- | --- | --- |

| Age, years | 35 (25, 46) | 38 (31, 44) | 0.10 |
| --- | --- | --- | --- |
| Sex=Female (vs Male) | 14 (36%) | 28 (53%) | 0.11 |
| Unable to work | 15 (38%) | 23 (43%) | 0.64 |
| History of mining | 6 (19%) | 5 (14%) | 0.52 |
| Interval since TB diagnosis, days | 277 (172, 485) | 372 (286, 520) | 0.27 |
| HIV | 13 (33%) | 15 (29%) | 0.69 |
| BMI, kg/m² | 20.5 (18.8, 22.4) | 20.7 (18.5, 22.9) | 0.34 |
| Underweight | 9 (23%) | 13 (25%) | 0.87 |
| Current/former tobacco smoker | 9 (23%) | 16 (30%) | 0.45 |
| Mental health | 16 (41%) | 25 (47%) | 0.56 |
| Memory difficulty | 11 (28%) | 24 (45%) | 0.10 |
| Cough | 3 (7.7%) | 26 (49%) | <0.001 |
| **Household comparators** | **N = 155** | **N = 130** |  |
| Age, years | 33 (24, 43) | 36 (25, 49) | 0.018 |
| Sex |  |  | 0.93 |
| Male | 46 (30%) | 38 (29%) |  |
| Female | 109 (70%) | 92 (71%) |  |
| Unable to work | 0 (0%) | 0 (0%) |  |
| History of mining | 6 (3.9%) | 7 (5.4%) | 0.51 |
| HIV | 29 (19%) | 32 (25%) | 0.23 |
| BMI, kg/m² | 24.1 (21.0, 28.5) | 24.1 (20.8, 28.3) | 0.71 |
| Underweight | 5 (3.2%) | 9 (7.0%) | 0.12 |
| Current/former tobacco smoker | 24 (15%) | 16 (12%) | 0.45 |
| Mental health | 46 (30%) | 52 (40%) | 0.071 |
| Memory difficulty | 34 (22%) | 35 (27%) | 0.34 |
| Cough | 18 (12%) | 24 (18%) | 0.11 |
| **Footnotes**: presented as number (percentage) or median (interquartile range). * Wald p values from logistic regression. | | | |

## Table G: Characteristics of households included in this analysis

| Characteristic | N = 192 |
| --- | --- |
| Crowding | 52 (27%) |
| Household income <2.15USD per person per day* | 158 (87%) |
| Household income per person per day in USD | 0.70 (0.41, 1.48) |
| Moderate-severe food insecurity (FIES) | 69 (38%) |
| Use of biomass fuels | 23 (12%) |
| **Footnotes**: Reported as median (interquartile range) or number (percentage). * Household income missing for 11 households and Food Insecurity Experience Scale (FIES) missing for 2 households.^9^ 2.15 United States Dollars per person per day corresponds to the 2022 international poverty line as defined by the World Bank.^10^ | |

## Figure B: Age distribution stratified by people with recent TB (n=96) and household comparators (n=285)

## Table H: Odds ratios for TB by demographics or health behaviours, adjusted for age and sex

| Characteristic | |  | Household comparators  N = 285 | People with recent TB  N = 92 | OR (95%CI) |
| --- | --- | --- | --- | --- | --- |
| **Demographics** | |  |  |  |  |
| **Gender** | | Men | 84 (29%) | 50 (54%) | – |
|  |  | Women | 201 (71%) | 42 (46%) | – |
| **Age, years** | |  | 34 (24–46) | 38 (28–44) | – |
| **School attainment*** | | None or primary | 40 (15%) | 13 (15%) | *Ref* |
|  |  | At least secondary | 230 (85%) | 73 (85%) | 0.73 (0.35–1.51) |
| **Medical aid cover** | | No | 259 (91%) | 86 (95%) | *Ref* |
|  |  | Yes | 25 (8.8%) | 5 (5.5%) | 0.50 (0.17–1.49) |
| **Currently pregnant†** | | No | 167 (91%) | 35 (95%) | *Ref* |
|  |  | Yes | 17 (9.2%) | 2 (5.4%) | 0.57 (0.12–2.66) |
| **History of work in mining** | | No | 272 (95%) | 57 (84%) | *Ref* |
|  |  | Yes | 13 (4.6%) | 11 (16%) | 2.14 (0.89–5.14) |
| **History of migration to South Africa** | | No | 237 (83%) | 55 (81%) | *Ref* |
|  |  | Yes | 48 (17%) | 13 (19%) | 1.02 (0.51–2.07) |
| **History of imprisonment** | | No | 277 (97%) | 61 (90%) | *Ref* |
|  |  | Yes | 8 (2.8%) | 7 (10%) | 2.06 (0.64–6.56) |
| **Health behaviours** | |  |  |  |  |
| **Current tobacco smoker‡** | | No | 257 (90%) | 87 (95%) | *Ref* |
|  |  | Yes | 28 (9.8%) | 5 (5.4%) | 0.24 (0.10–0.57) |
| **Current use of other tobacco products** | | No | 273 (96%) | 90 (98%) | *Ref* |
|  |  | Yes | 11 (3.9%) | 2 (2.2%) | 0.48 (0.10–2.25) |
| **Alcohol use category (AUDIT)** | Non-drinker | | 194 (68%) | 57 (62%) | *Ref* |
|  | Non-harmful drinking | | 58 (20%) | 21 (23%) | 0.89 (0.50–1.58) |
|  | Harmful/dependent | | 33 (12%) | 14 (15%) | 0.68 (0.33–1.40) |
| **AUDIT score§** | |  | 5 (2–11) | 4 (3–14) | 0.98 (0.94­–1.02) |
| **Use of other substances** | | No | 238 (86%) | 65 (74%) | *Ref* |
|  |  | Yes | 40 (14%) | 23 (26%) | 1.42 (0.77–2.62) |
| **Symptoms** | |  |  |  |  |
| **Cough** | | No | 243 (85%) | 63 (68%) | *Ref* |
|  |  | Yes | 42 (15%) | 29 (32%) | 2.58 (1.44–4.62) |
| **TB symptom screen positive** | | No | 240 (84%) | 62 (67%) | *Ref* |
|  |  | Yes | 45 (16%) | 30 (33%) | 2.47 (1.39–4.39) |
| **Footnotes**: presented as number (percentage) or median (interquartile range). Odds ratios are from logistic regression models, adjusted for age (4-level categorical variable: 18–29, 30–39, 40–49 and 50+ years) and sex, with TB status as the outcome. 95% confidence intervals (95%CI) use robust standard errors to account for household clustering. * Education status missing for 23 people. † Pregnancy reported among women only. ‡ 23% people with TB previously smoked, compared to 5% of comparators. § Alcohol use disorders identification test (AUDIT) score presented among people who reported drinking any alcohol. **¶** 4 people with TB and 3 household comparators who responded ‘don’t know’ to use of recreational drugs not shown in the table. Of 63 people who disclosed drug use, 29 (46%) reported use of cannabis, 5 reported use of methamphetamines and 2 people reported using cocaine. ** Duration of cough is among people reporting cough only. | | | | | |

## Table I: Symptoms among people with recent TB compared to household comparators

| **Characteristic** | **Household comparators**  N = 285 | **People with recent TB**  N = 92 |
| --- | --- | --- |
| **TB symptom screen positive*** | 45 (16%) | 30 (33%) |
| **Cough** | 42 (15%) | 29 (32%) |
| Duration of cough, weeks | 1.0 (0.4, 2.0) | 4.3 (1.0, 21.7) |
| **Productive cough** | 26 (9.1%) | 20 (22%) |
| Duration of productive cough, weeks | 0.9 (0.4, 1.3) | 3.1 (0.7, 14.1) |
| **Fever** | 4 (1.4%) | 7 (7.6%) |
| **Sweats** | 5 (1.8%) | 7 (7.6%) |
| **Weight loss** | 2 (0.7%) | 6 (6.5%) |

## Table J: Self-reported health and work status among people with recent TB

| **Characteristic*** | **N = 92** |
| --- | --- |
| **Health today compared to TB diagnosis** |  |
| Better | 67 (73%) |
| The same/worse | 25 (27%) |
| **Return to work** |  |
| Yes, returned to work | 61 (67%) |
| No, not returned to work due to poor health | 7 (7.7%) |
| No, not returned to work due to loss of employment | 5 (5.5%) |
| No, not working prior to diagnosis | 14 (15%) |
| No, not working due to other reason | 4 (4.4%) |
| **Footnotes**: presented as number (percentage). * Data missing for one person. | |

## Table K: Unadjusted proportion of people with chronic conditions, and associated measures, among people with recent TB and household comparators (N=377)

| **Characteristic** | **Household comparators**  N = 285 | **People with recent TB**  N = 92 |  |
| --- | --- | --- | --- |
| **HIV** | 61 (21%) | 28 (31%) |  |
| Currently on ART* | 44 (77%) | 25 (89%) |  |
| **Diabetes** | 25 (8.8%) | 11 (12%) |  |
| HbA1c, % | 5.80 (5.50, 6.10) | 5.60 (5.40, 6.00) |  |
| **Hypertension** | 98 (34%) | 18 (20%) |  |
| **Systolic BP, mmHg** | 118 (106­–128) | 126 (115–138) |  |
| **Diastolic BP, mmHg** | 77 (72–84) | 81 (75–88) |  |
| **Common mental health disorders**** | 98 (34%) | 41 (45%) |  |
| SSQ score | 5.0 (2.0, 8.0) | 6.0 (3.0, 9.0) |  |
| SSQ red flags present | 40 (14%) | 24 (26%) |  |
| Large impact of mental health symptoms | 51 (18%) | 30 (33%) |  |
| **Memory impairment** | 69 (24%) | 35 (38%) |  |
| **Underweight** | 14 (4.9%) | 22 (24%) |  |
| BMI category |  |  |  |
| Underweight | 14 (4.9%) | 22 (24%) |  |
| Healthy | 148 (52%) | 60 (65%) |  |
| Overweight | 74 (26%) | 6 (6.5%) |  |
| Obese | 48 (17%) | 4 (4.3%) |  |
| BMI, kg/m^2^ | 24.1 (20.8, 28.4) | 20.7 (18.7, 22.8) |  |
| **Anaemia** | 27 (9.5%) | 9 (9.8%) |  |
| Haemoglobin, g/dL | 14.0 (13.0–15.0) | 14.0 (13.0–15.2) |  |
| **Vision impairment** | 89 (31%) | 25 (27%) |  |
| **Impaired lung function†** | 33 (21%) | 18 (46%) |  |
| Pattern of impairment |  |  |  |
| None | 122 (79%) | 21 (54%) |  |
| Obstruction | 3 (1.9%) | 1 (2.6%) |  |
| Restrictive pattern | 30 (19%) | 17 (44%) |  |
| FEV1 Z score | -0.15 (-0.82, 0.58) | -1.49 (-2.11, -0.40) |  |
| FVC Z score | -0.40 (-1.35, 0.35) | -1.46 (-2.51, -1.08) |  |
| FEV1/FVC Z score | 0.68 (0.00, 1.40) | 0.64 (-0.40, 2.05) |  |
| **≥1 chronic condition** | 223 (78%) | 80 (87%) |  |
| **Multimorbidity**‡ | 137 (48%) | 47 (51%) |  |
| Number of conditions | 1.0 (1.0, 2.0) | 2.0 (1.0, 3.0) |  |
| **Footnotes**: * The percentage currently on ART is based on those with known HIV status (57 household comparators and 28 people with TB) and excludes people with newly diagnosed HIV during the health check. ** Red flags are two questions within the screening tool aimed at eliciting suicidal ideation or hallucinations. Participants were asked to self-report the impact of their mental health symptoms on daily life, in addition to the validated mental health questionnaire. † Data available for 155 household comparators and 39 people with TB. Obstruction defined as FEV1/FVC ratio Z score <-1.65 and restrictive pattern as FVC Z score <-1.65 with normal FEV/FVC ratio. None is defined as neither of these patterns. ‡ Multimorbidity defined as two or more of HIV, diabetes, hypertension, underweight, anaemia, common mental health disorder, vision impairment, impaired lung function; those with missing data were assumed not to have impaired lung function. **Abbreviations**: ART = anti-retroviral therapy, BMI = body mass index, BP = blood pressure, SSQ = Shona symptom questionnaire, TB = tuberculosis. | | | |

## Table L: Multimorbidity with and without self-reported memory difficulty (primary and sensitivity analyses)

|  | **Primary**  **(including memory difficulty)** | | | **Sensitivity**  **(excluding memory difficulty)** | | |
| --- | --- | --- | --- | --- | --- | --- |
| **Outcome** | **Comparators** | **Recent TB** | **aOR (95%CI)** | **Comparators** | **Recent TB** | **aOR (95%CI)** |
| ≥1 chronic condition | 231 (81%) | 84 (91%) | 2.30  (1.03–5.11) | 223 (78%) | 80 (87%) | 1.70  (0.83–3.49) |
| Multimorbidity (≥2 conditions) | 154 (54%) | 57 (62%) | 1.55  (0.86–2.78) | 137 (48%) | 47 (51%) | 1.18  (0.68–2.06) |

**Footnotes**: n (%) and age/sex-adjusted odds ratio (recent TB vs comparators), household cluster-robust standard errors.

## Table M: Association between reported memory impairment and age category, stratified by recent TB or not (post-hoc analysis)

|  | **Household comparators** (N=285) | | | **People with recent TB** (N=92) | | |
| --- | --- | --- | --- | --- | --- | --- |
|  | **No** | **Yes** | **aOR (95%CI)*** | **No** | **Yes** | **aOR (95%CI)*** |
| 18–29 years | 84 (38.9%) | 23 (33.3%) | *Ref* | 18 (31.6%) | 8 (22.9%) | *Ref* |
| 30–39 years | 63 (29.2%) | 9 (13.0%) | 0.52 (0.23–1.17) | 18 (31.6%) | 9 (25.7%) | 1.25 (0.37–4.10) |
| 40–49 years | 38 (17.6%) | 19 (27.5%) | 1.80 (0.84–3.85) | 13 (22.8%) | 10 (28.6%) | 1.85 (0.55–6.20) |
| 50+ years | 31 (14.4%) | 18 (26.1%) | 2.11 (0.98–4.54) | 8 (14.0%) | 8 (22.9%) | 2.20 (0.61–7.97) |
| **Footnotes**: * Odds ratios (OR) from logistic regression model for the outcome of memory impairment, adjusted for sex and household level clustering (using robust standard errors). CI = confidence interval. No and yes correspond to answers to the single question screening about memory impairment (‘In the past 12 months, have you had difficulty remembering things or following a story or conversation?’). | | | | | | |

## Table N: Association between reported memory impairment and presence of symptoms of mental health disorder

|  | **Household comparators** (N=285) | | | **People with recent TB** (N=92) | | |
| --- | --- | --- | --- | --- | --- | --- |
|  | **No** | **Yes** | **aOR (95%CI)*** | **No** | **Yes** | **aOR (95%CI)*** |
| SSQ -ve | 156 (83.4%) | 31 (16.6%) | *Ref* | 35 (68.6%) | 16 (31.4%) | *Ref* |
| SSQ +ve | 60 (61.2%) | 38 (38.8%) | 2.97 (1.64–5.37) | 22 (53.7%) | 19 (46.3%) | 2.23 (0.89–5.57) |
| **Footnotes**: * Odds ratios (OR) from logistic regression model for the outcome of memory impairment, adjusted for sex, age and household level clustering (using robust standard errors). CI = confidence interval. No and yes correspond to answers to the single question screening about memory impairment (‘In the past 12 months, have you had difficulty remembering things or following a story or conversation?’). | | | | | | |

## Table O: Self-reported memory difficulty among participants screening negative for symptoms of mental-health disorder (SSQ-negative)

| **Group** | **Memory difficulty, n (%)** | **aOR (95%CI)** | **p value** |
| --- | --- | --- | --- |
| Household comparators | 31/187 (17%) |  |  |
| Person with recent TB | 16/51 (31%) | 2.64 (1.17–5.94), | p=0.019 |

**Footnotes**: Adjusted odds ratio (aOR - recent TB vs comparators) and 95% confidence intervals (95%CI) for memory difficulty adjusted for age and sex, robust standard errors

## Table P: Odds of chronic conditions among people with recent TB compared to household comparators, adjusted for age, sex and body mass index (BMI; kg/m^2^)

| Characteristic | N=377* | aOR + BMI† | p |
| --- | --- | --- | --- |
| **Multimorbidity** |  |  |  |
| **≥1 chronic condition** | 303 | 1.40 (0.68–2.87) | 0.37 |
| **Multimorbidity*** | 184 | 0.70 (0.37–1.32) | 0.27 |
| **Infectious diseases** |  |  |  |
| **HIV** | 89 | 1.78 (1.00–3.15) | 0.05 |
| **Non-communicable diseases** |  |  |  |
| **Diabetes**‡ | 36 | 2.00 (0.91–4.37) | 0.08 |
| **Hypertension** | 116 | 0.54 (0.26–1.12) | 0.09 |
| **Memory impairment** | 104 | 1.98 (1.05–3.73) | 0.03 |
| **Common mental health disorders** | 139 | 1.23 (0.75–2.02) | 0.42 |
| **Vision impairment** | 114 | 0.58 (0.29–1.18) | 0.13 |
| **Impaired lung function** | 51 | 2.52 (1.10–5.75) | 0.03 |
| **Nutritional disorders** |  |  |  |
| **Underweight** | – | – | – |
| **Anaemia**‡ | 36 | 1.17 (0.50–2.74) | 0.72 |
| **Footnotes:** * Number with condition; other than for impaired lung function where denominator 194. † Odds ratios (and 95% confidence intervals) adjusted for age (4-level categorical variable: 18–29, 30–39, 40–49 and 50+ years), sex and body mass index (4-level categorical variable: underweight, healthy, overweight and obese, using World Health Organization definitions). ‡ Linear term for age and BMI used in these models to avoid over-parameterisation. Models of underweight were not adjusted for BMI and thus are not presented. | | | |

## Table Q: Proportion of prevalent disease that was undiagnosed among people with recent TB and household comparators (N=377)

| **Chronic conditions** | **Household comparators**  N = 285 | **People with recent TB**  N = 92 |
| --- | --- | --- |
| **HIV** | 4/61 (6.6%) | 0/28 (0.0%)* |
| **Diabetes** | 20/25 (80.0%) | 9/11 (81.8%) |
| **Hypertension** | 48/98 (49.0%) | 10/18 (55.6%) |
| **Mental health disorders** | 95/98 (96.9%) | 38/41 (92.7%) |
| **Vision impairment** | 60/89 (67.4%) | 19/25 (76.0%) |
| **Impaired lung function**† | 33/33 (100.0%) | 18/18 (100.0%) |
| **Underweight** | 14/14 (100.0%) | 22/22 (100.0%) |
| **Anaemia** | 27/27 (100.0%) | 9/9 (100.0%) |
| **Footnotes**: * Among people with TB, 15 (53%) reported that they were diagnosed with HIV at the same time as receiving their TB diagnosis. † Data on presence of impaired lung function not available for all participants due to missing spirometry data. | | |

## Table R: Prevalence and age-adjusted odds ratios for key outcomes, stratified by sex

| **Outcome** | **Household comparators** | **People with recent TB** | **aOR (95% CI)** |
| --- | --- | --- | --- |
| **Men** |  |  |  |
| HIV | 10/83 (12%) | 16/49 (33%) | 3.23 (1.31–7.92) |
| Hypertension | 23/84 (27%) | 7/50 (14%) | 0.38 (0.14–1.00) |
| Underweight | 10/84 (12%) | 17/50 (34%) | 4.70 (1.60–13.79) |
| Impaired lung function | 9/46 (20%) | 12/25 (48%) | 4.62 (1.36–15.71) |
| Memory difficulty | 18/84 (21%) | 15/50 (30%) | 1.63 (0.69–3.90) |
| Mental health | 25/84 (30%) | 24/50 (48%) | 2.10 (1.00–4.42) |
| ≥1 chronic condition | 67/84 (80%) | 47/50 (94%) | 4.13 (1.08–15.79) |
| Multimorbidity | 36/84 (43%) | 31/50 (62%) | 2.39 (1.09–5.22) |
| **Women** |  |  |  |
| HIV | 51/201 (25%) | 12/41 (29%) | 1.21 (0.56–2.61) |
| Hypertension | 75/201 (37%) | 11/42 (26%) | 0.41 (0.16–1.06) |
| Underweight | 4/200 (2%) | 5/42 (12%) | 6.82 (1.72–27.05) |
| Impaired lung function | 24/109 (22%) | 6/14 (43%) | 2.71 (0.83–8.92) |
| Memory difficulty | 51/201 (25%) | 20/42 (48%) | 2.68 (1.29–5.55) |
| Mental health | 73/201 (36%) | 17/42 (40%) | 1.18 (0.58–2.41) |
| ≥1 chronic condition | 164/201 (82%) | 37/42 (88%) | 1.55 (0.53–4.56) |
| Multimorbidity | 118/201 (59%) | 26/42 (62%) | 1.01 (0.44–2.32) |

**Footnotes**: adjusted odds ratio (aOR) and 95% confidence intervals (95%CI) for people with recent TB vs comparators within each sex, adjusted for age (4-level, or linear for impaired lung function, underweight) with robust standard errors for household-level clustering.

## Table S: Breakdown of EQ-5D components among people with recent TB and household comparators

| Characteristic | | Household comparators  N = 284* | People with recent TB  N = 91* | P† |
| --- | --- | --- | --- | --- |
| Anxiety/depression | None | 222 (78%) | 54 (59%) | 0.002 |
|  | Slight | 50 (18%) | 31 (34%) |  |
|  | Moderate | 8 (2.8%) | 3 (3.3%) |  |
|  | Severe | 4 (1.4%) | 2 (2.2%) |  |
|  | Unable | 0 (0%) | 1 (1.1%) |  |
| Mobility | None | 264 (93%) | 79 (87%) | 0.074 |
|  | Slight | 14 (4.9%) | 7 (7.7%) |  |
|  | Moderate | 3 (1.1%) | 5 (5.5%) |  |
|  | Severe | 2 (0.7%) | 0 (0%) |  |
|  | Unable | 1 (0.4%) | 0 (0%) |  |
| Pain/discomfort | None | 213 (75%) | 60 (66%) | 0.054 |
|  | Slight | 63 (22%) | 23 (25%) |  |
|  | Moderate | 6 (2.1%) | 7 (7.7%) |  |
|  | Severe | 2 (0.7%) | 1 (1.1%) |  |
|  | Unable | 0 (0%) | 0 (0%) |  |
| Usual activities | None | 273 (96%) | 84 (92%) | 0.046 |
|  | Slight | 6 (2.1%) | 5 (5.5%) |  |
|  | Moderate | 0 (0%) | 2 (2.2%) |  |
|  | Severe | 3 (1.1%) | 0 (0%) |  |
|  | Unable | 2 (0.7%) | 0 (0%) |  |
| Self-care | None | 277 (98%) | 85 (93%) | 0.12 |
|  | Slight | 4 (1.4%) | 5 (5.5%) |  |
|  | Moderate | 2 (0.7%) | 1 (1.1%) |  |
|  | Severe | 0 (0%) | 0 (0%) |  |
|  | Unable | 1 (0.4%) | 0 (0%) |  |
| Footnotes: * EQ-5D data was missing for 2 people. † P value from Fisher’s exact test. | | | | |

## Table T: Association between recent TB and health related quality of life or physical activity using ordinal logistic regression (Sensitivity analyses)

|  |  | **HHC**  (N=284)* | **TB**  (N=91)* | **OR (95% CI)** | **p** |
| --- | --- | --- | --- | --- | --- |
| **EQ-5D value** | None | 178 (63%) | 39 (43%) | 2.33 (1.45–3.75) | <0.001 |
|  | Slight | 84 (30%) | 40 (44%) |  |  |
|  | ≥Moderate | 22 (7.7%) | 12 (13%) |  |  |
|  | Median value | 0.90 (0.85, 0.90) | 0.86 (0.84, 0.90) |  |  |
| **IPAQ-SF** | Inactive | 45 (16%) | 17 (19%) | 1.01 (0.61–1.69) | 0.96 |
|  | Minimally active | 80 (28%) | 17 (19%) |  |  |
|  | HEPA active | 159 (56%) | 57 (63%) |  |  |
|  | Median MET min/week | 3,580 (1,483, 7,521) | 4,800 (1,271, 9,586) |  |  |
| **Footnotes**: * EQ%D missing for 2 participants. CI = confidence interval, HHC= household comparators, HEPA = health-enhancing physical activity, IPAQ-SF = International Physical Activity Questionnaire Short Form category of physical activity (categorised according to analysis guidelines^8^), MET = metabolic equivalents, OR = odds ratio, p = p value from likelihood ratio test, TB = people with recent TB. | | | | | |
